# Effects of Exercise Interventions on Estimated Pulse Wave Velocity and Mean Arterial Pressure in Overweight Adults: The Role of Modality

**DOI:** 10.31083/j.rcm2504139

**Published:** 2024-04-08

**Authors:** Sara Alghanim, Maha F. Alablani, Ali Alqutami, Rawan T. Alotaibi, Hyun Chul Jung, Lee Stoner, Abdullah B. Alansare

**Affiliations:** ^1^Department of Exercise Physiology, College of Sport Sciences and Physical Activity, King Saud University, 80200 Riyadh, Saudi Arabia; ^2^Department of Sports Coaching, College of Physical Education, Kyung Hee University-Global Campus, 17014 Yongin-si, Republic of Korea; ^3^Department of Sport and Exercise, University of North Carolina, Chapel Hill, NC 27599, USA

**Keywords:** HIIT, MICT, arterial stiffness, blood pressure, vascular health

## Abstract

**Background:**

Estimated pulse wave velocity (ePWV) is mathematically calculated from age and mean arterial pressure (MAP). We examined the effects of high-intensity interval training (HIIT) vs. moderate-intensity continuous training (MICT) on ePWV and MAP in insufficiently active overweight adults.

**Methods:**

Using the randomized controlled trial design, thirteen males (27.46 ± 3.80 years old; body mass index (BMI) = 29.61 ± 5.52) randomly completed either two-week HIIT (n = 7) or MICT (n = 6). HIIT consisted of 8 sessions of cycling, 20 min/session with an exercise-to-rest ratio of 10/50 s at ≥90% peak heart rate (${\mathrm{HR}}_{\text{peak}}$). MICT consisted of 8 cycling sessions, 40 min/session at 60–75% ${\mathrm{HR}}_{\text{peak}}$. Oscillometric brachial MAP was measured pre- and post-intervention, and ePWV was calculated. Two-way repeated measure analysis of variance examined the effects of time, intervention, and their interactions on ePWV and MAP.

**Results:**

Significant time effects were observed for ePWV and MAP, where both measures comparably decreased over time in HIIT and MICT groups (*p* < 0.05 for all). However, no significant intervention or interaction effects were detected, indicating no superiority of either exercise modality for ePWV or MAP improvements.

**Conclusions:**

This study uniquely revealed that two weeks of HIIT or MICT resulted in significant, comparable, and clinically meaningful decreases in ePWV and MAP among insufficiently active overweight adults. As such, overweight adults who have time as a constraint to engage in traditional exercise (i.e., MICT) can accomplish comparable vascular benefits by performing HIIT.

## 1. Introduction

Vascular stiffness, which exemplifies the degradation of the elasticity property of the vessel, is a major hallmark of vascular aging [1]. It has been recognized as a significant clinical predictor of cardiovascular disease (CVD) and mortality [1]. Moreover, it can provide clinically relevant information about CVD beyond the information achieved by traditional CVD risk factors [2]. As such, research and clinical practices have shown growing appeals for measuring vascular stiffness [2, 3, 4].

The current gold standard measure of vascular stiffness is through the carotid-femoral pulse wave velocity (cfPWV) [2, 4]. This method estimates the time transit of pressure waves produced by the myocardial contraction during systole and travels along the arterial tree [5]; faster cfPWV indicates stiffer vessels. Although cfPWV is a noninvasive and safe measure, it has drawbacks. For example, the cfPWV measurement typically requires expensive equipment and extensive training [6], thereby increasing the measurement burden in research and clinical settings. As a result, alternative methods of measuring vascular stiffness are necessary.

Pertinently, recent research has estimated cfPWV (ePWV) by using a mathematical equation that includes age and mean arterial pressure (MAP) [7]. This estimated pulse wave velocity (ePWV) has the same predictive value as cfPWV for CVD, particularly among healthy adults [7]. Subsequent studies have found that ePWV, by itself, can predict atrial fibrillation, CVD mortality, and all-cause mortality [8, 9]. Furthermore, ePWV has significant associations with various measures of vascular aging, including carotid intima-media thickness, carotid elastic modulus, and aortic augmentation index [10]. Yet, ePWV captured different vascular stiffness responses compared to cfPWV in an experimental research setting [11]. Despite this, ePWV remains significant in capturing clinically meaningful vascular stiffness impacts in research and clinical practices, regardless of its parallel responses or correlation with cfPWV.

Of particular interest, robust evidence implies that aerobic exercise can combat increased vascular stiffness. For example, previous systematic reviews and meta-analyses have reported beneficial influences of moderate-intensity continuous training (MICT) on vascular stiffness, measured by cfPWV, in children, adolescents, and adults with different characteristics [12, 13, 14, 15, 16]. Although the positive effects of MICT on cardiovascular health, including cfPWV, manifest [17], adults tend to perform insufficient amounts of MICT [18], with time being frequently self-reported as a constraint [18, 19, 20]. Alternatively, high-intensity interval training (HIIT) has been proposed as an exercise modality that is time efficient [21, 22] and more enjoyable [23] with a comparable or better influence on cardiovascular disease risk factors [24] and vascular stiffness (i.e., cfPWV) [13, 16, 25] compared to MICT in a variety of populations, including overweight and obese adults. Yet, the effects of HIIT and MICT on ePWV have not been explored.

As ePWV is emerging as a valuable cardiovascular measure and HIIT is being recommended for its superiority, it is important to examine the effectiveness of HIIT compared to other exercise modalities on ePWV. Therefore, using data from our previously published randomized controlled trial [21], the purpose of this secondary analysis was (1) to explore and compare the effects of HIIT vs. MICT on ePWV in insufficiently active overweight adults and (2) to explore the superiority effect of HIIT vs. MICT on MAP in insufficiently active overweight adults. It was hypothesized that HIIT would have a superior desirable effect on ePWV and MAP compared to MICT.

## 2. Materials and Methods

### 2.1 Study Procedure and Experimental Design

The current investigation was a secondary analysis of a previously published randomized controlled trial examining the effects of HIIT vs. MICT on cardiac autonomic functions in overweight adults [21]. Briefly, the original study was designed to assess the effects of two weeks (i.e., 8 sessions) of HIIT vs. MICT on blood pressure (BP) and time- and frequency-domain variables of heart rate variability (HRV) in overweight adults. The participants were randomly assigned to perform 8 HIIT or MICT training sessions over two weeks using a cycling ergometer (Monark 828E, Monark, Sweden). The simple randomization technique allocated each participant to their training group. Two pieces of paper were carefully folded after writing only the following on one side: “1” (indicates HIIT training) or “2” (indicates MICT training). The HIIT training was performed at an intensity of ≥90% of peak heart rate (${\mathrm{HR}}_{\text{peak}}$) with a 1:5 ratio for active and recovery cycling, respectively (i.e., 10 s active cycling at a speed of ≥100 rotation per minute (RPM) and 50 s recovery cycling at a speed of <50 RPM). Each HIIT session lasted for 20 min. Alternatively, the MICT training was performed at an intensity of 60 to 70% ${\mathrm{HR}}_{\text{peak}}$ at a speed of 60 RPM for 40 min/session. Regardless of the training modality, participants performed a five-minute warm-up and cool-down at an intensity of 40% ${\mathrm{HR}}_{\text{peak}}$ during each cycling session. All participants were instructed not to consume caffeine or perform heavy exercises for at least 24 hours before the measurement visits. This study’s procedure was reviewed and approved by the Institutional Review Board at the University of Louisiana at Monroe (No. 739-2016). All participants voluntarily participated and provided an informed consent form.

The results of the original study revealed that both HIIT and MICT comparably improved systolic BP and several time-domain variables of HRV. However, only HIIT significantly enhanced frequency-domain variables of HRV, suggesting the superiority of HIIT over MICT. For the current secondary analysis, we utilized BP values to calculate ePWV for both training groups, as described below.

### 2.2 Peak Heart Rate Determination

${\mathrm{HR}}_{\text{peak}}$ was determined by performing a graded exercise test on a cycle ergometer (Monark 828E, Monark, Varberg, Sweden). Participants started the test by cycling at a load of 50 watts for two minutes before a 25-watt load was added every two minutes until exhaustion. During the entire cycling period, participants were instructed to sustain the pedaling speed at 60 RPM and were verbally encouraged. Before starting, an HR monitor (Polar T31TM transmitter, Polar Electro, Kempele, Finland) was placed on the participants’ chests to measure their HR every minute of the test. Moreover, the rate of perceived exertion (RPE) was monitored every two minutes using the Borg scale (6 to 20 scale with a higher score indicating higher exertion). Furthermore, oxygen uptake was monitored throughout the cycling test using a metabolic cart (Quark CPET, Cosmed, Italy). ${\mathrm{HR}}_{\text{peak}}$ was verified if participants were: (1) unable to maintain the pedaling speed at 60 RPM for more than 5 s with verbal encouragement, (2) reaching a respiratory exchange ratio of >1.15, (3) reaching RPE of ≥19, and/or (4) reaching volitional exhaustion.

### 2.3 Participants

This study enrolled individuals who met the following inclusion criteria: male, with insufficient physical activity (i.e., self-reported <150 min/week of moderate-intensity physical activity by using the short version of the International Physical Activity Questionnaire), adults between 20 to 40 years old, and able to cycle on an ergometer [21]. The exclusion criteria included having an issue affecting the participants’ ability to cycle (e.g., knee and lower back pain) or having any cardiovascular disease. All included participants provided informed consent.

### 2.4 Anthropometric Measurements

Participants’ body weight (kg) was measured to the nearest 0.01 kg using a body weight scale (1.40 LCD Display, Walgreens, Deerfield, IL, USA). In addition, participants’ body height (in cm) was also measured to the nearest 0.01 cm using a drop-down tape measure (439 Detecto, Webb City, MO, USA). These values were then used to calculate participants’ body mass index (BMI) by using the following equation: BMI = body’s weight (kg)/body’s height (meter squared ($m^{2}$)).

Seventeen participants met the inclusion criteria and were enrolled in the study (HIIT = 9; MICT = 8). However, two participants from each intervention group withdrew for personal reasons before completing their eighth session. Thus, the total number of participants who completed all sessions and were included in the current analyses was 13 (HIIT = 7; MICT = 6). The general characteristics of the participants included were compared, and the results are displayed in Table 1. Overall, the young adult participants had normal blood pressure but were overweight, with no significant differences between the HIIT and MICT groups.

**Table 1.** **Baseline characteristics of the participants (n = 13)**.

Characteristic	HIIT group (n = 7)	MICT group (n = 6)	*t* (*p*-value)
	(Mean ± SD)	(Mean ± SD)	
Age (year)	26.00 ± 2.77	29.17 ± 4.36	–1.59 (0.140)
Height (cm)	174.43 ± 10.10	173.25 ± 7.33	0.24 (0.827)
Weight (kg)	90.68 ± 22.24	88.37 ± 14.85	0.22 (0.833)
BMI (${\mathrm{kg/m}}^{2}$)	29.57 ± 5.55	29.65 ± 6.02	–0.025 (0.981)
SBP (mmHg)	118.00 ± 3.94	119.00 ± 10.48	–0.235 (0.818)
DBP (mmHg)	70.86 ± 6.57	72.92 ± 3.37	–0.69 (0.504)
MAP (mmHg)	90.11 ± 2.01	92.64 ± 6.51	–0.98 (0.348)
ePWV (m/s)	6.43 ± 0.09	6.60 ± 0.38	–1.202 (0.255)

Abbreviations: BMI, body mass index; cm, centimeter; DBP, diastolic blood pressure; ePWV, estimated pulse wave velocity; SBP, systolic blood pressure; *t*, independent *t*-test values; kg, kilogram; ${\mathrm{kg/m}}^{2}$, kilogram per meter squared; MAP, mean arterial pressure; mmHg, millimeter of mercury; m/s, meter per second; SD, standard deviation; HIIT, high-intensity interval training; MICT, moderate-intensity continuous training.

### 2.5 Blood Pressure Measurement

During pre- and post-training interventions, morning oscillometric systolic (SBP) measurements and diastolic blood pressure (DBP) measurements were performed between 08.00 and 09.00 AM. An electronic sphygmomanometer (Advantage 6021, American Diagnostic Corporation, Hauppauge, NY, USA) was used to complete these measurements. First, participants quietly sat for five minutes on a chair with their feet on the floor and back and arms supported. Thereafter, two SBP and DBP measurements were performed, separated by one-minute intervals. Notably, our laboratory examined the reliability of these BP measurements and found good to excellent reliability for SBP and DBP (intraclass correlation coefficient (ICC) = 0.89 and 0.93, respectively). Averages of the two SBP and DBP measurements were then utilized to calculate MAP: MAP = DBP + 1/3 (SBP – DBP).

### 2.6 Estimated Pulse Wave Velocity Calculation

To calculate pre and post-ePWV for each training intervention, the following formula was utilized: ePWV = 9.587 – (0.402 × age) + (4.560 × ${\mathrm{10}}^{-3}$× ${\mathrm{age}}^{2}$) – (2.621 × ${\mathrm{10}}^{-5}$× ${\mathrm{age}}^{2}$× MAP) + (3.176 × ${\mathrm{10}}^{-3}$× age × MAP) – (1.832 × ${\mathrm{10}}^{-2}$× MAP) [7].

### 2.7 Statistical Analysis

Participants’ age, anthropometric, and BP measurements are reported as the mean and standard deviation. The two groups (i.e., HIIT and MICT) were compared using independent tests to examine any baseline differences. The Shapiro–Wilk test was used to determine the normality of the ePWV and MAP data. Moreover, Levene’s test was utilized to check the homogeneity of the ePWV and MAP data. To examine the effects of time, training intervention (HIIT vs. MICT), and their interaction (time x training intervention), two-way repeated measure analysis of variance (ANOVA) tests were performed. The significant level was set as *p* ≤ 0.05. All statistical analyses were completed using JASP software (JASP 0.16.4 Version, Amsterdam, Netherlands).

## 3. Results

**Fig. 1.**
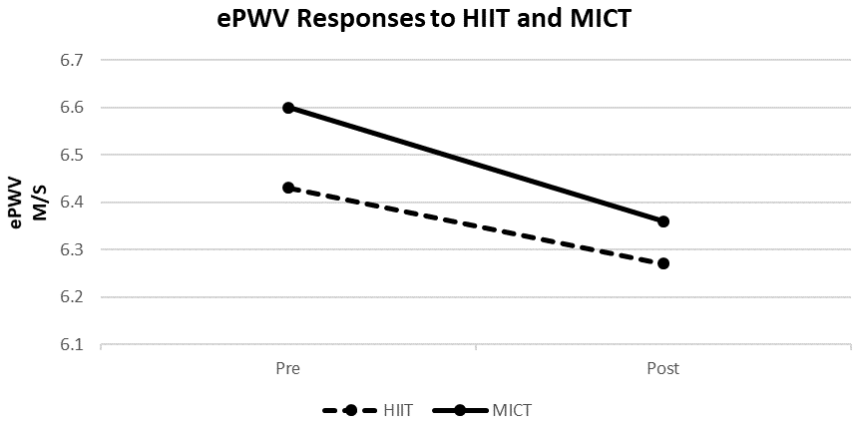
**Effects of HIIT (n = 7) vs. MICT (n = 6) on resting estimated pulse wave velocity**. HIIT, high-intensity interval training; MICT, moderate-intensity continuous training; M/S, meter per second; ePWV, estimated pulse wave velocity.

**Fig. 2.**
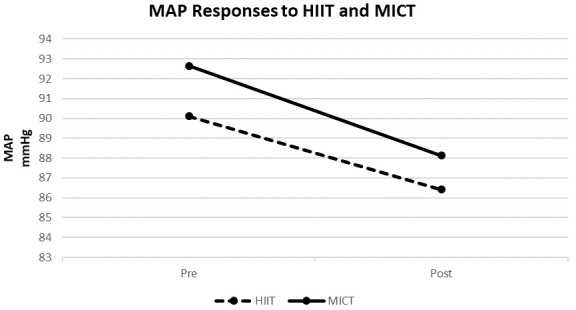
**Effects of HIIT (n = 7) vs. MICT (n = 6) on resting mean arterial pressure**. HIIT, high-intensity interval training; MICT, moderate-intensity continuous training; MAP, mean arterial pressure; mmHg, millimeter of mercury.

Table 2 and Figs. 1,2 compare the effects of the two weeks of HIIT vs. MICT training (8 sessions) on resting ePWV and MAP. The main effects were observed on the resting ePWV and MAP (*p* < 0.05), indicating that both training interventions significantly benefitted these outcomes over time. However, the effect of training intervention and its interaction with time suggests no differences between the effects of HIIT and MICT on ePWV and MAP (*p* > 0.05 for all).

**Table 2.** **Effects of HIIT (n = 7) vs. MICT (n = 6) on resting estimated pulse wave velocity and mean arterial pressure**.

Outcome	Group	Pre (mean ± SD)	Post (mean ± SD)	∆ (pre minus post)	Condition effect F (*p*-value)	Time effect F (*p*-value)	Time x condition F (*p*-value)
ePWV (m/s)	HIIT	6.43 ± 0.09	6.27 ± 0.22	–0.2	0.39 (0.544)	8.75 (**0.013**)	1.11 (0.315)
	MICT	6.60 ± 0.38	6.36 ± 0.29	–0.2			
MAP (mmHg)	HIIT	90.11 ± 2.01	86.41 ± 5.14	–3.7	0.08 (0.786)	7.77 (**0.018**)	0.86 (0.374)
	MICT	92.64 ± 6.51	88.12 ± 5.13	–4.5			

Abbreviations: MAP, mean arterial pressure; mmHg, millimeter of mercury; m/s, meter per second; n, number of participants; ePWV, estimated pulse wave velocity; SD, standard deviation; F, F statistics; m/s, meter per second; HIIT, high-intensity interval training; MICT, moderate-intensity continuous training. Bold indicates a significant effect (*p* < 0.05).

## 4. Discussion

This unique secondary analysis was performed first to explore the effects of HIIT vs. MICT on ePWV. Further, the superiority of HIIT compared to MICT for improving MAP was evaluated. We revealed novel findings that two weeks of HIIT or MICT resulted in significant and clinically meaningful decreases (favorable effects) in ePWV and MAP in insufficiently active overweight adults. Yet, no interaction effects for a time by the intervention were observed for either outcome, suggesting no superiority of either exercise modality.

Previous studies (n = 10) assessed the influence of HIIT vs. MICT for ≥4 weeks on cfPWV in various adult populations. The results of these studies were recently summarized in a systematic review and meta-analysis [25]. Though individual studies observed mixed findings (i.e., favor HIIT, favor MICT, no effects), the pooled estimate showed no significant differences between HIIT and MICT for cfPWV improvements [25]. In harmony with this observation, we detected comparable significant effects of HIIT and MICT on ePWV in overweight adults with no evidence supporting the superiority of HIIT (*p*-value for the interaction effect >0.05). As such, the current evidence suggests the lack of superiority of HIIT compared to MICT for vascular stiffness improvements (i.e., neither cfPWV nor ePWV) following aerobic exercises in adults.

Noteworthy, the difference in ePWV (pre- minus post-intervention) that we observed over time in the HIIT and MICT groups was –0.2 m/s (for both). This ePWV reduction would clinically correlate to 10.6%, 6.2%, and 7% decreases in the risks for hemorrhagic stroke, ischemic stroke, and total stroke, respectively [9]. Furthermore, a reduction of 0.2 m/s in the ePWV would relate to 4.6% and 5.2% reduced risk for all-cause mortality and cardiovascular mortality, respectively [26]. Although, altogether, no superiority by either exercise modality was found, our results indicate that both HIIT and MICT can provide clinically meaningful protective influences on vascular stiffness measured by ePWV in insufficiently active overweight adults.

Subsequently, the influence of HIIT vs. MICT on MAP in adults was also investigated. A recent study reported significant reductions in MAP following six weeks of HIIT (–4.8 mmHg) and MICT (–2.7 mmHg) in insufficiently active men who are overweight or obese [27]; however, no significant interaction effects were noted for the time by which the intervention was observed. In agreement with these findings, we noticed significant decreases in MAP following two weeks of HIIT (–3.7 mmHg) and MICT (–4.5 mmHg) without significant interaction effects in insufficiently active overweight adults. Importantly, these reductions established in MAP (–2.7 to –4.8 in the current and previous studies [27]) would be associated with 2.7% to 4.8% lower risk of major cardiovascular events, including coronary and cerebrovascular events [28]. Thus, although existing evidence indicates no superiority of HIIT vs. MICT for MAP boosting, both exercise modalities can afford clinically desirable influence on MAP.

While the current study did not observe the superior influence of HIIT to MICT on either ePWV or MAP in insufficiently active overweight adults, previous studies reported seemingly contradicting results on several other cardiovascular variables. For instance, an earlier randomized controlled trial found the superior influence of HIIT to MICT on reverse left ventricular remolding, end-diastolic, and end-systolic volume in CVD patients [29]. In contrast, a systematic review and meta-analysis included seven randomized controlled trials that detected no significant differences between the effects of HIIT vs. MICT on either systolic or diastolic blood pressure in patients with pre-to-established hypertension [30]. Although reasons for these discrepancies are not clearly understood, potential explanations may include characteristics of participants (e.g., CVD patients, apparently healthy, overweight/obesity, interindividual variability, hemorheological profile) or exercise protocols (e.g., 2 weeks vs. more than 4 weeks, treadmill vs. cycling) [31, 32]. For example, recent evidence suggests that overweight adults with pre-hypertension exhibit significant differences in BP responses to HIIT compared to normotensive individuals [31]. Hence, there is a need for further well-designed randomized controlled trials that consider important factors such as the prevalence of positive effects on interventions or hemorheological profile to acquire a more comprehensive understanding of the impact of HIIT vs. MICT on cardiovascular health.

Although this study has multiple strengths, such as using a randomized controlled trial design and the gold standard measure of BP assessment, a few limitations should be considered when interpreting our findings. First, both HIIT and MICT interventions lasted for two weeks only. Though this time course was found to significantly boost vascular function [33, 34], a more recent report suggested that vascular adaptation to exercise interventions appeared to peak after eight weeks of exercise interventions [35]. As such, the currently observed improvements in ePWV and MAP may reflect the instant, but not long-lasting, effects of HIIT and MICT. Therefore, future studies comparing the influence of eight weeks of HIIT vs. MICT on ePWV are needed to reach more comprehensive conclusions on the influence of different exercise modalities on ePWV. Furthermore, the sample in the current study was relatively small after a few dropouts (n = 13). This small sample size might have reduced the statistical power, affecting the strength of our findings. Thus, further studies with larger sample sizes are warranted to confirm the currently reported results.

### Clinical Significance

We confirm the beneficial influence of different exercise modalities on vascular stiffness measured by ePWV and MAP. Uniquely, we reveal that both HIIT and MICT have comparable, clinically meaningful effects on ePWV and MAP in insufficiently active overweight adults. These findings could have implications for promoting exercise for better vascular health, particularly among individuals who report a lack of time. Performing HIIT is time-saving and enjoyable and can lead to desirable vascular improvements.

## 5. Conclusions

In short, this secondary analysis evaluated the effects of HIIT vs. MICT on ePWV and MPA in insufficiently active overweight adults. We have uniquely revealed that both HIIT and MICT significantly improved ePWV and MAP, with no evidence for the superiority of either exercise modality. Remarkably, these findings suggest that two weeks of HIIT and MICT can provide a clinically meaningful influence on ePWV and MAP, such that the risk of CVD and mortality decreases by large proportions. As such, overweight adults with time constraints for engaging in traditional exercise (i.e., MICT) can accomplish comparable vascular benefits by performing HIIT.

## Data Availability

The datasets used and/or analyzed during the current study are available from the corresponding author on reasonable request.

## Abbreviations

BMI, body mass index; BP, blood pressure; CVD, cardiovascular disease; DBP, diastolic blood pressure; ePWV, estimated pulse wave velocity; HIIT, high-intensity interval training; MAP, mean arterial pressure; MICT, moderate-intensity continuous training; SBP, systolic blood pressure.
